# Self-reported health behaviors and longitudinal cognitive performance in late middle age: Results from the Wisconsin Registry for Alzheimer’s Prevention

**DOI:** 10.1371/journal.pone.0221985

**Published:** 2020-04-23

**Authors:** Kimberly D. Mueller, Derek Norton, Rebecca L. Koscik, Martha C. Morris, Erin M. Jonaitis, Lindsay R. Clark, Taylor Fields, Samantha Allison, Sara Berman, Sarah Kraning, Megan Zuelsdorff, Ozioma Okonkwo, Nathaniel Chin, Cynthia M. Carlsson, Barbara B. Bendlin, Bruce P. Hermann, Sterling C. Johnson

**Affiliations:** 1 Wisconsin Alzheimer’s Institute, School of Medicine and Public Health, University of Wisconsin–Madison, Madison, WI, United States of America; 2 Wisconsin Alzheimer’s Disease Research Center, School of Medicine and Public Health, University of Wisconsin–Madison, Madison, WI, United States of America; 3 Department of Communication Sciences and Disorders, University of Wisconsin–Madison, Madison, WI, United States of America; 4 Department of Biostatistics and Medical Informatics, University of Wisconsin–Madison, Madison, WI, United States of America; 5 Rush Institute for Healthy Aging, Rush University Medical Center, Chicago, IL, United States of America; 6 Department of Neurology, University of Wisconsin–Madison, Madison, WI, United States of America; 7 Geriatric Research and Education Center, William S. Middleton Memorial Veteran’s Hospital, Madison, WI, United States of America; Nathan S Kline Institute, UNITED STATES

## Abstract

**Background:**

Studies have suggested associations between self-reported engagement in health behaviors and reduced risk of cognitive decline. Most studies explore these relationships using one health behavior, often cross-sectionally or with dementia as the outcome. In this study, we explored whether several individual self-reported health behaviors were associated with cognitive decline when considered simultaneously, using data from the Wisconsin Registry for Alzheimer’s Prevention (WRAP), an Alzheimer’s disease risk-enriched cohort who were non-demented and in late midlife at baseline.

**Method:**

We analyzed longitudinal cognitive data from 828 participants in WRAP, with a mean age at baseline cognitive assessment of 57 (range = 36–78, sd = 6.8) and an average of 6.3 years (standard deviation = 1.9, range = 2–10) of follow-up. The primary outcome was a multi-domain cognitive composite, and secondary outcomes were immediate/delayed memory and executive function composites. Predictors of interest were self-reported measures of physical activity, cognitive activity, adherence to a Mediterranean-style diet (MIND), and interactions with each other and age. We conducted linear mixed effects analyses within an Information-theoretic (IT) model averaging (MA) approach on a set of models including covariates and combinations of these 2- and 3-way interactions. The IT approach was selected due to the large number of interactions of interest and to avoid pitfalls of traditional model selection approaches.

**Results:**

Model-averaged results identified no significant self-reported health behavior*age interactions in relationship to the primary composite outcome. In secondary outcomes, higher MIND diet scores associated with slower decline in executive function. Men showed faster decline than women on delayed memory, independent of health behaviors. There were no other significant interactions among any other health behaviors and cognitive trajectories.

**Conclusions:**

When multiple covariates and health behaviors were considered simultaneously, there were limited weak associations with cognitive decline in this age range. These results may be explained alone or in combination by three alternative explanations: 1) the range of cognitive decline is in middle age is too small to observe relationships with health behaviors, 2) the putative associations of these health behaviors on cognition may not be robust in this age range, or 3) the self-reported measures of the health behaviors may not be optimal for predicting cognitive decline. More study may be needed that incorporates sensitive measures of health behaviors, AD biomarker profiles, and/or other disease comorbidities.

## Introduction

The estimated prevalence of dementia in America in 2019 was 5.8 million, and it is projected to rise to 13.9 million by 2050 if prevention or cures are not identified. Delaying the onset of dementia by five years would result in a financial savings of up to 40% in 2050 [1], as well as a reduction of emotional burden to families and caregivers. Consequently, there is considerable interest in identifying lifestyle modifications that may prevent or delay cognitive decline in aging adults, particularly those with elevated dementia risk.

Numerous observational studies have shown correlations between lifestyle activities, such as engagement in physical, cognitive, and social activities, and cognitive decline in aging adults [2,3]. Furthermore, in the last decade there have been multiple studies showing that consuming a Mediterranean-style diet, or aspects of this type of eating (e.g., fish), also confer a reduced risk of cognitive decline in aging adults [4–6]. Studies that examine multiple health behaviors and cognitive decline from late middle age–the time at which lifestyle interventions may be most effective–are lacking.

Although analyses using lifestyle modifications as predictors often control for factors that may influence cognition such as age, educational attainment, and intellectual ability, studies often include one main predictor of interest (e.g., cognitive activity), but not other lifestyle activities that may tend to co-occur [7]. Such studies do not explain to what degree one lifestyle behavior contributes to variability in cognition in interaction with other health behaviors. Moreover, advice that targets single health behaviors may have unintended consequences for modifying other health behaviors that tend to co-occur (e.g., increasing physical activity but engaging in poor diet) [8]. No studies, to our knowledge, have investigated the combined influences of several health behaviors plus covariates known to affect cognition on cognitive trajectories in late-middle-aged adults who are clinically unimpaired. However, there are problems when trying to examine multiple predictors within multiple models: namely, the possible inflation of Type I error due to multiple comparisons. Traditional statistical approaches, such as stepwise model selection, whereby the “best” fitting model is the final model selected, can result in overly optimistic results and conclusions, or can deem other predictors as unimportant when in fact model fits are similar [1]. Recently our group addressed such model-fitting issues when investigating the combined influences of sex, APOE genotype and literacy on early to late middle-aged adults using an “Information Theoretic Model Averaging” approach. This approach evaluates a set of plausible hypotheses and combines the relative strength of information across all considered models instead of selecting one single model. By using this approach, our group found that increased literacy was associated with better cognitive performance over time in an early to late-middle aged cohort (Koscik et al., 2019).

In the present study, our aims were to determine the combined influences of multiple health behaviors and multiple relevant covariates, while at the same time using a statistical approach that was rigorous and thorough, based on a set of hypotheses routed in the current health behaviors and aging literature. The hypotheses for this study were that one or more self-reported health behaviors—cognitive activity, physical activity and diet—would be associated with attenuated cognitive decline when considered simultaneously, using data from the Wisconsin Registry for Alzheimer’s Prevention (WRAP). Because we were interested in determining whether these three health behaviors (individually and together), in addition to age, sex and educational attainment (variables known to affect cognition) were associated with cognitive trajectories in a relatively young and unimpaired group, and thus there were multiple combinations of models with multiple predictors, we opted to use an Information Theoretic Model Averaging approach to determine the relative strength of each model.

## Methods

### Participants

WRAP is a longitudinal observational cohort enriched for a parental history of late-onset AD. Enrollment began in 2001 with follow-up assessments occurring every two years; for additional protocol details, see Johnson et al., 2018. [9] Due to this targeted enrollment, participants have a higher percentage of the *APOE-ε4* allele than the general population (~41%). At the time of these analyses, 1549 participants were enrolled, with a mean age at baseline of 53.7 (sd = 6.6), 72.6% had a parental history of sporadic AD, and all were free of dementia at baseline. To be included in these analyses, participants had to have at least two visits; be free at baseline of clinical Mild Cognitive Impairment (MCI) and histories of stroke, Parkinson’s disease, multiple sclerosis or epilepsy at any visit; and had to have complete data for the predictors (completed questionnaires for diet, physical activity, and cognitive activities (n = 828)). This study was conducted in compliance with the ethical principles for human subjects research defined in the Declaration of Helsinki, including approval by the University of Wisconsin–Madison Institutional Review Board.

### Cognitive outcomes

The full WRAP battery is described in greater detail in Johnson et al. (2018) [9]. For the primary outcome, we calculated a composite analogous to the Preclinical Alzheimer’s Cognitive Composite 4 (PACC4) described by Donohue et al. (2014) [10] based on our available test measures. Specifically, we computed a PACC4 composite as the average of standardized test scores (z-scores) of total recall learning trials 1–5 from the Rey Auditory Verbal Learning Test (RAVLT) [11], total scores for the Logical Memory II subtest (i.e., delayed recall of stories A and B) from the Wechsler Memory Scale—Revised (WMS-R) [12], total scores from the Digit Substitution test of the Wechsler Abbreviated Intelligence Scale-Revised (WAIS-R) [13], and the total score from the Mini-Mental Status Examination (MMSE) [14].

*Secondary outcomes*. We also averaged standardized test scores to obtain three domain-specific cognitive composites previously described by our group [15], including Immediate Recall (contributing tests: RAVLT total from trials 1–5; WMS-R LM Immediate Recall, stories A and B; and BVMT-R Immediate Recall, trials 1–3); Delayed Recall (contributing tests: RAVLT Long-Delay Free Recall, WMS-R LM Delayed Recall, and BVMT-R Delayed Recall); and Executive Functioning (contributing tests: Trail Making Test Part B total time to completion, Stroop Neuropsychological Screening Test, color-word interference total items completed in 120 seconds, and WAIS-R Digit Symbol Coding total items completed in 90 seconds. The z-score for Trail Making Test Part B was multiplied by -1 before inclusion in the composite so that higher z-scores indicated better performance for all tests).

### Predictors of interest

At each study visit, participants completed a comprehensive neuropsychological testing battery, completed detailed medical history and lifestyle questionnaires, and provided blood samples, vitals, and other objective and physical assessments described elsewhere [9]. The three main lifestyle predictors—diet, physical activity, and cognitive activity—were based on self-report using the scales described below (see Table 1). We used data obtained from the first visit at which all three predictors were available (median = visit 5), and cognitive data from all visits from which the composites were available (range = visits 2–6).

**Table 1:** Description of self-reported health behaviors.

Health Behavior	Self-Report Measure and Description
**Diet**	The Mediterranean-DASH Intervention for Neurodegenerative Delay Questionnaire (MIND; 15 items; Morris et al., 2015). The MIND diet score is based on consumption of 10 “brain healthy” food groups (e.g, leafy greens, nuts, berries, fish) and 5 unhealthy food groups (e.g., red meats, fried food, pastries/sweets). Range in WRAP: 3–14 (mean = 9.4, sd = 1.9)
**Physical Activity**	Participants completed the Women's Health Initiative physical activities questionnaire (McTieman et al., 2003). We calculated a total score of metabolic hours (MET) per week based on participants’ responses to questions about the frequency and duration of walking outside the home for >10 minutes without stopping, and engagement in mild (e.g., golf), moderate (e.g., calisthenics), and vigorous (e.g., jogging) exercise [18] For mild, moderate, and vigorous exercises, frequency ranged from none to ≥5 days/week and duration ranged from <30 minutes to ≥1 hour/session. Range in WRAP: 0–98 (mean = 18, sd = 15)
**Cognitive Activity**	The Florida Cognitive Activities Scale (FCAS) includes 25-items covering a range of cognitive activities (e.g., playing games, solving puzzles, cooking from new recipes) yielding a total cognitive activities score [20]. Range in WRAP sample: 14–70 (mean = 43.5, sd = 8.3)

#### Physical activity

Participants answered questions about their physical activity based on a subset of items from the Women’s Health Initiative Observational study [16]. These questions were administered at visit 2 and each longitudinal visit thereafter. Details about this questionnaire are described elsewhere [17,18]; in brief, we constructed composite recreational physical activity variables based on several questions. Specifically, participants were asked about frequency, intensity, and duration of walking outside the home for more than 10 minutes; frequency and duration of strenuous exercise (examples provided included aerobics, jogging, swimming laps), moderate-intensity activities (e.g., biking, easy swimming, folk dancing), and low-intensity activities (slow-dancing, bowling, golf). We converted these responses to hours exercised per week, assigned metabolic equivalent (MET) values for each of the three intensity activities, and multiplied the MET level by hours exercised per week, as was done in previous work [17–19], to obtain a variable “MET-hours per week.”

#### Cognitive activity

In order to quantify the type and frequency of engagement in cognitive activities, participants answered questions from the Florida Cognitive Activities Scale (FCAS) [20]. The FCAS is a 25-item scale that measures intensity, frequency and duration of a variety of activities differing in cognitive demand, including “playing chess, bridge or knowledge games,” “gardening,” and “preparing meals from new recipes.” [20] Responses were weighted according to frequency and duration and summed to create the total “cognitive activities” variable.

#### Diet

To measure dietary behavior, participants completed a 15-item questionnaire capturing adherence to a hybrid of the Mediterranean-style diet and the Dietary Approaches to Stop Hypertension (DASH) diet termed Mediterranean-DASH Intervention for Neurodegenerative Delay (MIND) [4,6]. The MIND diet score is comprised of 10 brain healthy foods (e.g., natural, plant-based foods, berries and green leafy vegetables, whole grains, fish, olive oil) and 5 unhealthy food groups (red meats, butter/margarine, cheese, pastries/sweets, fried/fast food). To obtain a total MIND score, the frequency of consumption of each category was summed following guidelines published by Morris et al (2015) [6]; due to updates in the MIND questionnaire, coding varied slightly from the original publication (for details, see S1A Table for the revised MIND questionnaire and S2B Table for the coding decisions that differ from the original).

#### Other covariates

Covariates included age, sex, *APOE-ε4* risk score (a summative log-odds ratio of all *APOE* alleles [21]), and Wide Range Achievement Test– 3 (WRAT-3) Reading subtest standard score [22]. The WRAT-3 Reading subtest was used as a proxy for educational attainment, as previous literature suggests that reading level may be a more sensitive proxy for educational experience than self-reported years of education [23–25].

#### Sensitivity analysis

Because our Health Behavior predictors are time-invariant (e.g., we used data from the first time point at which all three health behaviors were available to create the predictor, median visit = 5), we performed sensitivity analyses to determine if, within our data, scores on the FCAS or MIND diet tended to change significantly from one visit to the next. We examined n = 149 participants who had two time-points of FCAS and MIND data, and plotted the raw values of each time point versus one another with their first principle component (i.e., Pearson correlation line), and also performed non-parametric bootstrapping of the first principal component’s slope over 10,000 iterations. For the FCAS measure, the slope of the linear trend was 1.02 with a correlation of .743 between responses at the two time points; for MIND diet the slope of the linear trend was 1.15, with a correlation of .664. Bootstrapped 95% confidence intervals confirmed that the difference between the responses for FCAS time 1 vs. time 2, and MIND time 1 vs. time 2, were not statistically different from zero.

### Data analysis

The FCAS and MIND questionnaires were added to the study after baseline; accordingly, health behavior measures were obtained from the first visit at which all health behavior data was available for a participant (median = visit 5). That is, the set of health behavior predictors are cross-sectional (time-invariant), while the cognitive outcome measures are longitudinal.

Given our goal of characterizing the combined influences of multiple health behavior predictors and related interactions, we opted to use an information theoretic (IT) modeling technique detailed by our group in previous work. [26] In brief, the IT framework evaluates a set of plausible scientific hypotheses, and uses the relative strengths of information considered across all models to obtain model-averaged parameter estimates, instead of selecting a single model based on traditional model selection techniques. In so doing, after fitting all of the models of interest to each outcome (with differing combinations of health behavior predictors and interactions), results are combined across models in proportion to the relative strength of information each model contributes [26]. The IT framework also offers advantages over a traditional model selection approach (e.g., forward or backward selection), in that it allows comparison of fits across all models and reduces over-estimation of effect sizes compared to standard models.

#### The model set

Based on previous research indicating that physical activity, cognitive activity, and diet modified associations between time and cognition, we developed a set of 9 linear mixed-effects models to investigate in these analyses. Model 1 included the previously mentioned set of covariates; additional models built on Model 1 and incorporated various combinations of the 3 health behavior variables and interactions with age (the longitudinal covariate in our models). We used the age at each visit as the time variable in order to account for differences in baseline ages and time intervals between visits. We centered age at the average of all visits for ease of interpretation, with precision to two decimal places for improved temporal resolution.

For each outcome and each model in the set, we fit the model using a linear mixed effects structure (random effects were subject-specific intercepts). Health behavior predictors were converted to z-scores (mean = 0, sd = 1) for analyses. For each of the four cognitive outcomes modeled, model diagnostics were performed on the best fitting model for that outcome in the set of 9 models (lowest AICc value in the set). No major concerns were noted in any of the best fitting models for outliers, heteroscedasticity, residual trends, non-normality of residuals / random effects, trends between random effects and residuals.

#### Model outputs

After fitting the models, model statistics were extracted, and the minimum Akaike’s Information Criterion-corrected (AICc) statistics were used to calculate the relative strength of information between each model and the best fitting model, which we then used to calculate model weights (smaller AICcs have larger weights). Regression coefficient results from each model were multiplied by their corresponding weights, and weighted results were summed together for the final model averaged result. Nonparametric bootstrapping of this process over 10,000 replicates was used to create 95% confidence intervals of the model averaged coefficients, using the quantile method; confidence intervals that contain zero are interpreted as non-significant. In addition to obtaining the model-averaged parameter estimates, we used the model log-likelihoods to conduct likelihood ratio tests of each of models 2–9 vs model 1 to test whether the addition of multiple behavior terms improved model fit over the covariates-only model. In exploratory/secondary results, we compare parameter estimates from the best fitting model with the model-averaged parameter estimates for an outcome only if the likelihood ratio tests for that model vs model 1 was significant at the α = 0.05 level.

All analyses were performed in R version 3.4.0. Linear mixed effect regressions were fit using ‘lmer’ in the lme4 package; AICc-based model statistics were calculated using ‘aictab’ in the AICcmodavg package; bootstrapping was performed using HTCondor version 8.6.3.

## Results

### Sample characteristics

Sample characteristics at visit 2 (when all cognitive measures for the PACC were first available) are shown in Table 2. Mean(sd) age was 57.7(6.4) years, 67.6% were female, average years of education was 16.2(2.7) and 98.1% were non-Hispanic white. For demographic and clinical characteristics of those participants who were excluded due to having cognitive data only at one time point, see S1 Table.

**Table 2:** Sample demographic and health characteristics at Wave 2 data collection.

	n = 828
Age at first cognitive composite* (mean (sd))	57.8 (6.4)
Years of Follow-up (mean (sd, median, range))	6.3 (sd = 1.9, median = 7, range = 2–10)
Sex = Female (%)	560 (67.6)
*APOE-ε4* Carrier (%)	312 (37.7)
WRAT-3 Reading—Standard Score (mean (sd))	105.7 (9.3)
CES-D Depression Score (mean (sd))	6.6 (6.7)
Race (n, % non-Hispanic White)	812 (98.2)
Years of Education (mean (sd))	16.2 (2.8)
Systolic Blood Pressure (mean (sd))	124.2 (15.9)
Diastolic Blood Pressure (mean (sd))	74.1 (9.6)
Total Cholesterol (mean (sd))	200.3 (37.7)
Body Mass Index (mean (sd))	28.7 (6.1)
Waist-Hip Ratio (mean (sd))	0.86 (0.10)
MMSE (mean (sd), median)	29 (.9), 30
AVLT Total (mean (sd))	51.1 (8.4)
Logical Memory Story Recall Delayed (mean (sd))	26.2 (6.9)
Digit Symbol Coding	57.5 (10.2)

Abbreviations: APOE Risk Score: a summative odds ratio of all APOE alleles (Darst et al., 2017); WRAT-3 –Wide Range Achievement Test (Wilkinson, 1984); CES-D–Center for Epidemiology Scale for Depression. MMSE: Mini-Mental State Examination (Folstein et al., 1975); AVLT: Rey-Auditory-Verbal Learning Test (Schmidt et al. 1996).

*The Wave 2 visit is “baseline” for cognition in these analyses as it was the first visit at which the analyzed composites could be calculated.

### IT framework results

**Fig 1:**
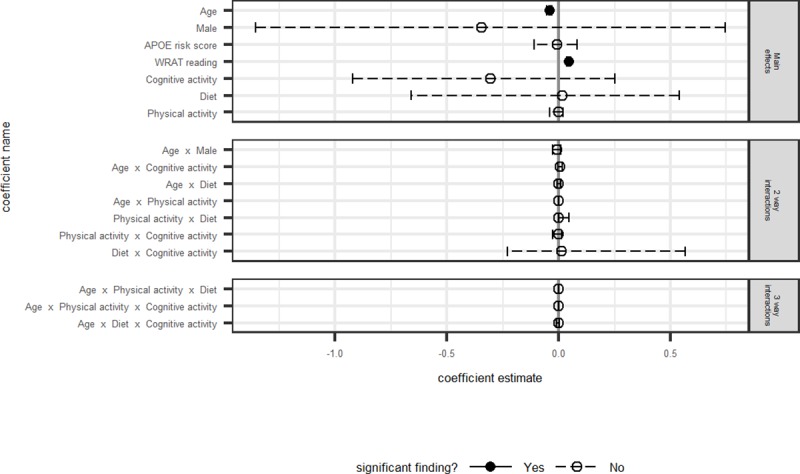
PACC: Model averaged regression coefficient estimates and 95% confidence intervals.

**Fig 2:**
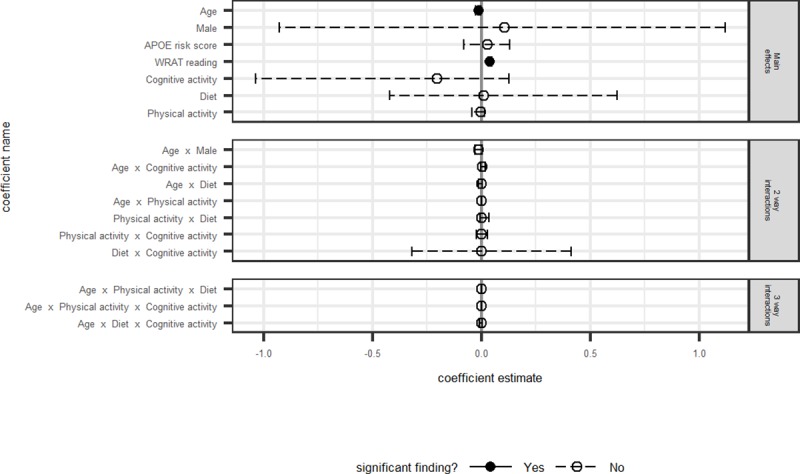
Immediate memory: Model averaged regression coefficient estimates and 95% confidence intervals.

**Fig 3:**
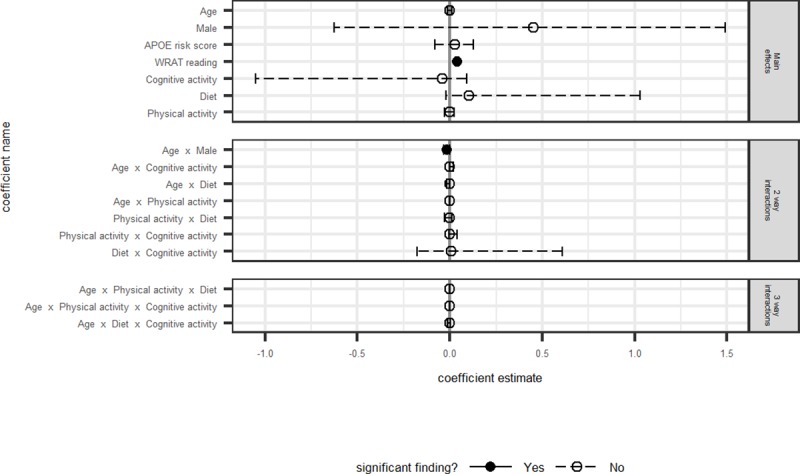
Delayed memory: Model averaged regression coefficient estimates and 95% confidence intervals.

**Fig 4:**
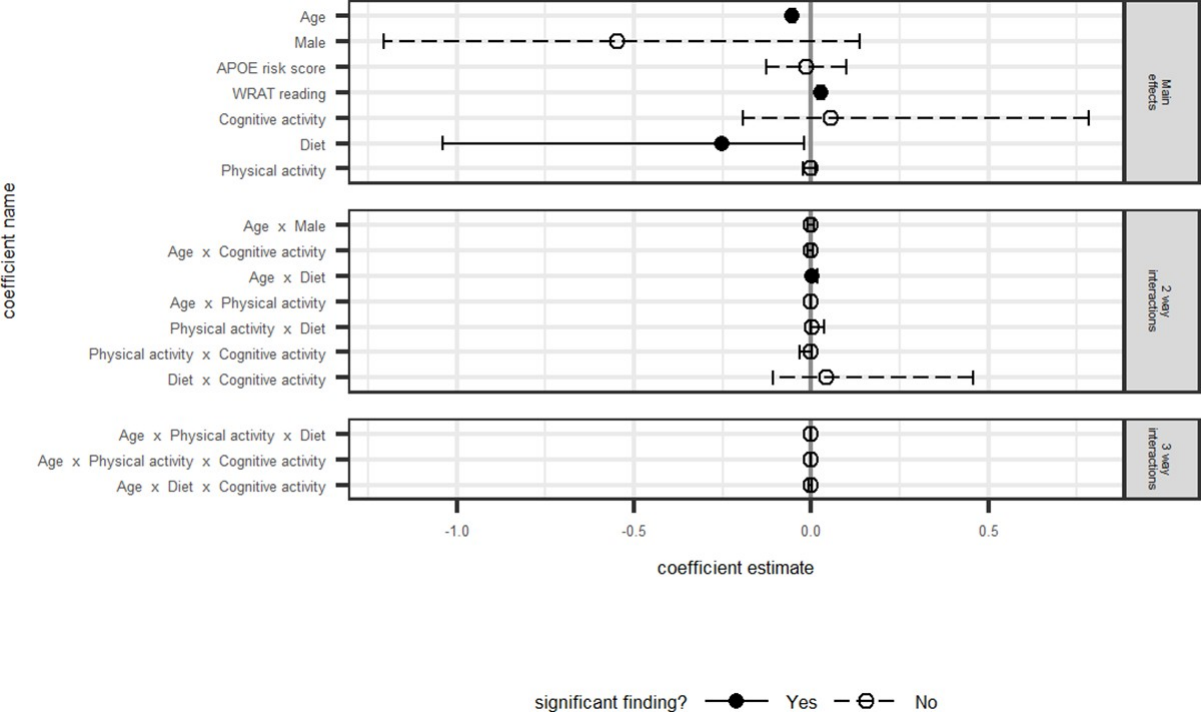
Executive function: Model averaged regression coefficient estimates and 95% confidence intervals.

Model fit statistics, differences between AICc and the best model’s AICc, model weights, log-likelihoods, and likelihood ratio tests are presented in Table 3 for each outcome. Model averaged coefficients and corresponding 95% confidence intervals for all non-intercept terms are depicted in Figs 1–4. Results are discussed by outcome below.

**Table 3:** Model fit statistics, AICc, model weights, log-likelihood, and likelihood ratio tests.

		**PACC4**							**EXECUTIVE FUNCTION**						
**MODEL #**	**Highest order terms**	**K**	**AICc**	**ΔAICc**	**AICc Wt**	**LL**	LRT*	p-value*	**K**	**AICc**	**ΔAICc**	**AICc Wt**	**LL**	**LRT**	p-value*
**M1**	Age, Sex, APOE, WRAT-3, random effects	8	7586.53	2.5	0.17	-3785.24			8	5238.86	2.11	0.11	-2611.4		
**M2**	Age* Physical activity	10	7589.98	5.95	0.03	-3784.95	0.58	0.748	10	5241.85	5.1	0.02	-2610.89	1.02	0.600
**M3**	Age* Mind Diet	10	7589.91	5.87	0.03	-3784.92	0.64	0.726	10	5237.36	0.61	0.23	-2608.64	5.52	0.063
**M4**	Age * Cognitive Activity	10	**7584.03**	**0**	**0.6**	**-3781.98**	**6.52**	**0.038**	10	5237.81	1.06	0.18	-2608.87	5.06	0.080
**M5**	All 3 age* health behavior	14	7591.32	7.29	0.02	-3781.59	7.3	0.294	14	5240.11	3.35	0.06	-2605.98	10.84	0.093
**M6**	age* Phys. Activity * Mind Diet	14	7595.6	11.57	0	-3783.73	3.02	0.806	14	5240.36	3.6	0.05	-2606.11	10.58	0.102
**M7**	age * Phys. Activity * Cognitive Activity	14	7588.98	4.95	0.05	-3780.42	9.64	0.141	14	5241.98	5.23	0.02	-2606.92	8.96	0.176
**M8**	age * Mind Diet * Cognitive Activity	14	7587.64	3.61	0.1	-3779.75	10.98	0.089	14	**5236.75**	**0**	**0.31**	**-2604.3**	**14.2**	**0.027**
**M9**	All 3 3-way interactions from M6-M8	20	7595.83	11.8	0	-3777.77	14.94	0.245	20	5242.17	5.42	0.02	-2600.94	20.92	0.052
LRT = -2*(LL M1—LL MN), WHERE N = 2 THROUGH 9; P-VALUE OBTAINED FROM CHI-SQUARE WITH D.F. = K FOR MODEL N—K FOR M1															
		**Immediate Memory**							**Delayed Memory**						
**MODEL #**	Highest order terms	**K**	**AICc**	**ΔAICc**	**AICc Wt**	**LL**	LRT*	p-value*	**K**	**AICc**	**ΔAICc**	**AICc Wt**	**LL**	**LRT**	p-value*
**M1**	Age, Sex, APOE, WRAT-3, random effects	8	7787.05	0.61	0.35	-3885.50			8	**7573.6**	**0**	**0.52**	-3778.78		
**M2**	Age* Physical Activity	10	7790.09	3.65	0.08	-3885.01	0.98	0.613	10	7577.52	3.92	0.07	-3778.72	0.12	0.942
**M3**	Age* Mind Diet	10	7791.01	4.57	0.05	-3885.47	0.06	0.970	10	7575.32	1.72	0.22	-3777.62	2.32	0.313
**M4**	Age * Cognitive Activity	10	**7786.44**	**0**	**0.47**	-3883.18	4.64	0.098	10	7576.62	3.02	0.11	-3778.27	1.02	0.600
**M5**	All 3 age* health behavior	14	7792.98	6.55	0.02	-3882.42	**6.16**	0.406	14	7581.09	7.49	0.01	-3776.47	4.62	0.593
**M6**	age* Phys Activity * Mind Diet	14	7796.47	10.04	0	-3884.16	**2.68**	0.848	14	7582.34	8.74	0.01	-3777.1	3.36	0.762
**M7**	age * Phys Activity * Cognitive Activity	14	7793.42	6.98	0.01	-3882.64	**5.72**	0.455	14	7583.06	9.46	0	-3777.46	2.64	0.852
**M8**	age * Mind Diet * Cognitive Activity	14	7792.63	6.19	0.02	-3882.24	**6.52**	0.368	14	7578.18	4.58	0.05	-3775.02	7.52	0.275
**M9**	All 3 3-way interactions from M6-M8	20	7801.37	14.93	0	-3880.54	9.92	0.623	20	7587.51	13.91	0	-3773.61	10.34	0.586
LRT = -2*(LL M1—LL MN), WHERE N = 2 THROUGH 9; P-VALUE OBTAINED FROM CHI-SQUARE WITH D.F. = K FOR MODEL N—K FOR M1															

Abbreviations: AICc = Akaike’s Information Criteria-corrected; ΔAICc = difference in AICc between presented and best model; LL = log-likelihood; LRT = likelihood ratio test; PACC-4 = Preclinical Alzheimer’s Cognitive Composite Score-4; MA = Model-averaged; CI = 95% Confidence Interval; MIND = Mediterranean-DASH Intervention for Neurodegenerative Delay (Morris et al., 2015); FCAS = Florida Cognitive Activities Scale (Schinka et al., 2004)

### Primary outcome: PACC4

The overall rate of change in PACC-4 z-scores for the sample, without adjusting for practice effects was .006(.25); the lowest quartile of change in PACC-4 was .30(.20) standardized score units per year.

The model-averaged estimates shown in Fig 1 indicate that the main effects of age and WRAT-3 reading were significantly associated with the PACC4, such that participants with higher WRAT-3 reading and participants who were younger had better PACC4 scores. There were neither significant main effects nor significant interactions between age and any of the three modifiable behavior factors. In order to compare the model averaged results to the “best fitting model” as would be done in traditional model selection procedures, we evaluated the list of models individually. The best fitting model of the set was model 4 (Model 1, or base model (age, sex, WRAT-3 reading) plus cognitive activity (FCAS) and its interaction with age). The likelihood ratio test indicated that adding these terms resulted in a better fit than Model 1. Table 4 presents beta estimates and their confidence intervals for this model relative to the model-averaged parameter estimates. The model-averaged beta and confidence interval for the Cognitive Activity*age interaction were .006(-.002 - .02), compared with the model 4 beta and confidence interval of .007(.00003–0.01). Thus, although the model 4 beta and confidence interval did not contain zero, effects were weak, small, and similar to the IT model-averaged estimates.

**Table 4:** Beta estimates and 95% confidence intervals for best fitting models* relative to their model-averaged parameter estimates.

	PACC4		Executive Function	
Model Term	MA beta (CI)	Best model beta (CI)*	MA CI	Best model beta (CI)*
**APOE Risk**	-0.008 (-0.11–0.08)	-0.007 (-1.06–0.09)	-0.012 (-0.13–0.10)	-1.41e-02 (-1.31e-01 - .10)
**WRAT III Reading subtest**	0.046 (0.04–0.05)	0.0457 (3.75e-02-0.05)	0.028 (0.02–0.04)	2.81e-02 (1.87e-02–0.04)
**Age**	-0.042 (-0.05- -0.03)	-0.042 (-5.08e-02 - -0.03)	-0.053 (-0.06- -0.05)	-5.21e-02 (-5.79e-02 - -0.05)
**Sex**	-0.347 (-1.36–0.76)	-0.401(-1.37e+00 - .52)	-0.548 (-1.21–0.14)	-5.64e-01 (-1.19e-02–0.04)
Age*Sex	-0.008 (-0.26–0.01)	-0.007 (9–2.22e-02 - .01)	0 (-0.01–0.01)	-5.25e-05 (-9.91e-03–0.01)
**Met Hours per Week (Physical Activity)**	-0.001 (-0.04–0.02)	--	-0.001 (-0.02–0.01)	--
**MIND (Diet)**	0.018 (-0.66–0.54)	--	-0.252 (-1.04- -0.02)	-3.55e-01 (-6.53e-01 - -0.03)
**FCAS (Cognitive Activity)**	-0.306 (-0.92–0.25)	-0.397 (9–8.48e-01–0.064)	0.056 (-0.19–0.79)	1.15e-01 (-1.95e-01–0.44)
Physical Activity*age	0 (0.0–0.0)	--	0 (0.0–0.0)	--
MIND Diet*age	0 (-0.01–0.01)	--	0.004 (0.00–0.02)	5.18e-03 (7.28e-05–0.01)
Cognitive Activity*age	0.006 (-0.002–0.02)	0.007 (2.95e-05–0.01)	0 (-0.01–0.01)	-2.17e-01 (-5.15–0.005)
Physical Activity*MIND Diet*age	0 (-0.001–0.00)	--	0 (-0.00–0.00)	--
Physical Activitys*Cognitive Activity* age	0 (0.00–0.00)	--	0 (0.00–0.00)	--
MIND Diet *Cognitive Activity*age	0 (-0.01–0.003)	--	-0.001 (-0.01–0.00)	-3.00e-03 (-7.72e-03-0.002)

*"Best Model" presented only if LRT with M1 showed the model added significantly above covariates. PACC-4 best model = Model 4; Executive Function best model = Model 8.

Abbreviations: PACC-4 = Preclinical Alzheimer’s Cognitive Composite Score-4; MA = Model-averaged; CI = 95% Confidence Interval; MIND = Mediterranean-DASH Intervention for Neurodegenerative Delay (Morris et al., 2015); FCAS = Florida Cognitive Activities Scale (Schinka et al., 2004)

### Secondary outcomes

#### Immediate memory composite

Main effects of age and WRAT-3 Reading were significantly associated with Immediate Memory, such that younger participants or those with higher WRAT-3 Reading scores performed better on Immediate Memory (Fig 2). As with the PACC4, there were no significant main effects or interactions between the lifestyle behavior predictors of interest and the Immediate Memory outcome. The best fitting model of the set was model 4, which included the base model (age, sex, WRAT-3 reading, APOE Risk Score) plus Cognitive Activity and its interaction with age; the likelihood ratio test, however, was non-significant, indicating that the addition of these terms did not fit the data better than the base model including only age, sex, WRAT-3 reading, and APOE Risk (i.e., Model 1).

#### Delayed memory composite

None of the health behaviors were associated with delayed memory performance. The best fitting model of the set was model 1 (base model only).

Higher WRAT-3 Reading scores were significantly associated with higher delayed memory scores. The significant interaction between age and sex indicated faster annual decline in men than in women (Fig 3).

#### Executive function composite

**Fig 5:**
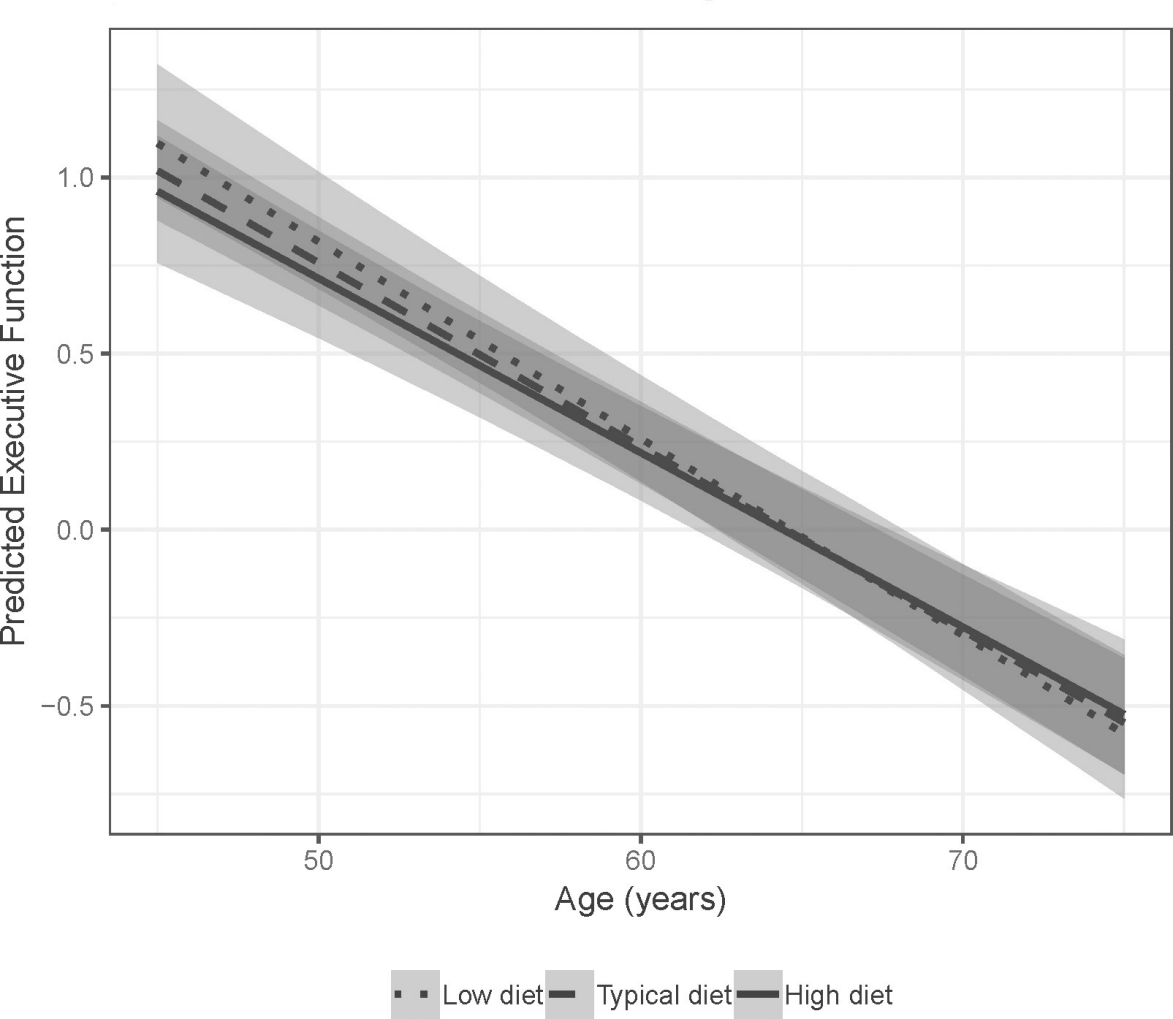
Model averaged predicted executive function performance by MIND diet with 95% confidence intervals.

The model averaged estimates shown in Fig 4 indicate that main effects of age and WRAT-3 Reading were associated with executive functioning, such that younger participants and those with higher WRAT-3 scores performed better on the composite. A significant interaction for diet and age suggested that those participants who reported diets more congruent with the MIND diet showed slower decline over time on executive function (Fig 5). No other predictors of interest were significantly associated with executive functioning. The best fitting model of the set was model 8, including the base model (age, sex, WRAT-3, APOE) plus the interaction between MIND Diet, Cognitive Activity, and age, as well as all corresponding lower-order terms. The likelihood ratio test indicated that this model fit the data better than base Model 1. Results from this model were consistent with the model averaged estimates and indicated small effect sizes. Specifically, using model-averaged parameter estimates and assuming a female participant with average Cognitive Activity, those with MIND Diet z-score = 1 had estimated annual decline of -.049 SDs per year, compared to -.057 SDs per year for those with MIND z-scores = -1. Using model 8 output, estimated annual change was -.047 vs -.057, respectively (for a woman with average Cognitive Activity). Table 4 presents beta estimates and their confidence intervals for this model relative to the model-averaged parameter estimates.

## Discussion

In this study, we examined whether multiple self-reported health behaviors–physical activity, cognitive activity, and diet–modified age-related cognitive trajectories in a group of cognitively unimpaired, late-middle-aged adults. General media narratives advise that such behaviors are protective, yet often the research behind these narratives has a singular focus (e.g., only diet or only physical activity). We used an information-theoretic model averaging approach so that we could include all three behaviors and multiple interactions at the same time, while reducing the risk of overfitting the data and at the same time effectively controlling for Type I error. We evaluated longitudinal outcomes in several cognitive domains that have been found to be sensitive to early cognitive decline, specifically; a global cognitive composite (Preclinical Alzheimer’s Cognitive Composite-4 [PACC4] [10]), immediate memory, delayed memory, and executive function). Our primary analysis failed to offer support that such behaviors influence cognitive decline in this cohort of late middle-aged people.

In this relatively large (n = 828) and young sample (mean age at visit 5 follow-up was 65, standard deviation = 6.3) using a comprehensive approach, we found no associations between longitudinal cognition and self-reported diet or exercise. Analysis of secondary outcomes revealed a small effect regarding diet and executive function. Specifically, those participants who reported more adherence to a Mediterranean-DASH Intervention for Neurodegenerative Delay (MIND) [4] diet showed slower decline on an executive function composite than those participants who reported less adherence to the MIND-style diet. The effect size for this interaction was quite small, despite the fact that several other studies have shown more robust associations between adherence to the MIND diet and reduced risk of cognitive decline and dementia [4–6]. One possible reason for this is the relatively young age of the cohort and corresponding reduced range of cognitive decline consistent with middle-agers. The annual rate of change in the sample is -.007. Mormino et al., 2017 analyzed rates of change in PACC scores in individuals who were amyloid positive/negative, and APOE-ε4 positive/negative, and found that, in a sample with a mean age of ~74, the average annual change in PACC for APOE-ε4 carriers was -.043, and APOE status was not a significant predictor of cognitive decline when accounting for amyloid positivity. Although the rate of change in the full WRAP sample is slower than that reported in Mormino et al., 2017 (-.007 vs. -.043), researchers from our group have identified a subset of individuals who are declining at a faster rate (see Johnson et al., 2018; Koscik et al., 2019). We have operationalized this subset of participants who are declining as “cognitively unimpaired-declining,” and the concept is akin to Stage 2 from the NIA-AA research framework for Alzheimer’s disease (Jack et al., 2018). When we examine the annual rate of change in the lowest negative quartile of change (i.e., the subset of participants potentially representing those with subclinical cognitive change) the mean change in PACC-4 was .30 (range = .13 to 1.40). Thus, although the group as a whole is showing a small amount of change, there is a subgroup of individuals who are declining at a faster rate. Therefore, it will be important to reexamine these associations as our sample ages and completes additional cognitive testing visits.

The lack of strong associations among health behaviors and longitudinal cognition in this late middle-aged cohort may also partially be explained by the fact that we did not consider objective measures of health (e.g., body mass index, blood pressure) or overall cardiovascular risk as covariates or mediating factors on cognition, as was done in other studies [27]. It is therefore possible that self-reported health behaviors alone may be of little consequence in late middle age to young old age, either due to problems with self-report, or a lack of medical management of comorbid conditions, or a combination of factors; future studies of individuals in late middle age that control for chronic disease and medical management of comorbidities, or examine cardiovascular risk as a mediating factor, will help provide a better understanding of these relationships. Second, we took a comprehensive approach to looking at health behaviors individually and when controlling for other health behaviors. Studies that have taken a similar approach have had mixed results. For example, Sturman et al. (2005) examined whether participation in physical activity slowed cognitive decline after accounting for participation in cognitively stimulating activities; although physical activity was associated with a slower rate of cognitive decline, when adjusting for participation in cognitive activities, depression and vascular disease, the observed association of physical activity with to cognitive decline was no longer statistically significant [7]. Because individuals who engage in cognitive activity may be more likely to engage in physical activity and/or healthy diets, it is important to continue to attempt to elucidate the respective contributions that each behavior may have on cognition in late middle age and beyond [8].

We used continuous measures of health behavior composite scores, similar to previous studies of these behaviors [28]. It is possible that categorical measures (e.g., tertiles indicating “high,” “medium”, and “low” engagement in behaviors) may provide added sensitivity, in that the discriminatory ability of these variable self-report measures to cognitive decline may lie in those who show the highest versus lowest engagement in behaviors [29]. Since it is likely that different health behaviors have an additive effect on health and possibly cognition, combining several health behaviors into an overall “health behavior index” may further elucidate these relationships. A recent large retrospective cohort study developed a weighted healthy lifestyle score, including no smoking, regular physical activity, healthy diet, and moderate alcohol consumption, which they categorized in to favorable, intermediate and unfavorable lifestyles. Using this approach, the authors found that a favorable lifestyle was associated with lower dementia risk, including in those participants with higher genetic risk for dementia [30].

It is important to consider the reliability of self-reported measures of lifestyle in a longitudinal cohort study such as WRAP. First, both the physical exercise composite and the MIND diet scores may either underestimate or overestimate actual engagement in these behaviors. The physical activity questions are broad (e.g., mild, moderate, strenuous) and participants may not have considered all possible sources of physical activity in their responses. Similarly, the 15-item revised MIND diet questionnaire had relatively few questions about food intake, including frequency of consumption of individual items, resulting in imprecise measurement of the food intake within each MIND diet component, therefore possibly underestimating the effect of diet on cognitive decline. Furthermore, some literature suggests that self-report may overestimate actual physical activity, possibly a result of effects such as social desirability bias [31]. The conflicting evidence about such measures suggests that self-reported health behaviors data should be interpreted with caution. Several large, multidomain intervention clinical trials are underway which will address the limitations of observational studies, including some issues with self-report of health and behavior, but most of these studies are enrolling individuals who are 5–10 years older than the WRAP cohort, and still may not inform how lifestyle behaviors affect cognition in late middle-age. Examples of such trials include the Finnish Geriatric Intervention Study to Prevent Cognitive Impairment and Disability (FINGER) study, a multi-domain intervention study implementing dietary advice, a physical exercise program, cognitive training, and advice regarding management of metabolic and vascular risk factors [32]. One recent report showed that dietary improvement was associated with slower rate of decline in executive function (mean age of intervention and control groups = 69.5) [33]. Examples of other health behavior clinical trials aimed at reducing or preventing cognitive decline include Exercise in Adults with Mild Memory Problems (EXERT) [34], Multidomain Alzheimer Preventive Trial (MAPT) [35], and the U.S. Study to Protect Brain Health Through Lifestyle Intervention to Reduce Risk (POINTER) [36].

Finally, it is possible that our sample is too young to see clinically meaningful associations between health behaviors and cognitive decline. For instance, while Morris et al. found that greater adherence to the MIND diet was associated with slower rates of decline in cognition [5], this group was considerably older (mean age at follow-up = 81) than our WRAP sample (mean age at follow-up = 65). Similarly, some of the most compelling studies showing associations between health behaviors in midlife and reduced risk of cognitive decline either had dementia as the outcome [3–6] or had older participant samples [3,28]. Furthermore, even though the cohort is enriched for *APOE-ε4* genotype (38% in this subset), our group has not found significant associations with the *APOE-ε4* allele and longitudinal cognition [9], nor did we see any associations with *APOE-ε4* and cognition in the present study, despite the fact that APOE is a noted risk factor for cognitive decline [37]. This discrepancy may also be due to the younger age of the WRAP cohort and the reduced range of cognitive decline. As Mormino et al., 2017 suggest, it is possible that the association between APOE and risk for cognitive decline may be due to the fact that *APOE-ε4* carriers are more likely to be amyloid positive than non-carriers, and that amyloid is driving the association, not *APOE*.

Our comprehensive information theoretic model averaging approach allowed us to test multiple predictors and multiple interactions, while avoiding issues common to model selection procedures (overfitting, anticonservative results, etc.). This methodological innovation lends credibility to our modest results. It should be noted that the WRAP sample is predominantly a self-selected family history cohort study, thereby enriched for AD-risk, and the majority of participants are highly educated, non-Hispanic white, and reside in the Upper Midwest, all of which may limit the generalizability of findings to the general population. Furthermore, we did not evaluate other social determinants of health that may affect access and ability to engage in health behaviors, such as neighborhood disadvantage, socioeconomic status, and other factors that may contribute to health disparities overall.

We used a time-invariant measurement of self-reported health behaviors (from median visit 5), and longitudinal cognition from visit 2 and beyond, due to the fact that the cognitive activity and MIND diet questionnaires were implemented late in the study, at median visit 5. As a result, we did not have the numbers of participants available to perform a longitudinal analysis of health behaviors, but still designed to use the >6 years of follow-up cognitive data. Therefore, we cannot rule out the possibility that reverse causation may obscure our results (i.e., cognitive decline may predict lower engagement in physical activity). When WRAP has acquired additional longitudinal data regarding health behaviors, it will be important to examine whether cognitive decline is associated with change in health behaviors (or vice-versa).

We did not examine whether or not these health behaviors had a protective effect on cognition in the context of disease (i.e., Alzheimer’s disease biomarkers including beta-amyloid plaques, phosphorylated tau, and neurodegeneration [38]), which will be an important area of future study in the WRAP cohort. In a 2015 study by Schultz and colleagues, higher cardiorespiratory fitness was found to modify the effects of amyloid burden on cognition, such that individuals with relatively high amyloid burden demonstrated better scores on cognitive testing if those individuals had high levels of cardiorespiratory fitness [39]. Additionally, Clark et al. (2019) found that hypertension and obesity moderated the relationship between cognitive decline and amyloid burden [40]. These results suggest that amyloid burden or other AD biomarkers may be an important variable to consider when assessing interactions between health behaviors and longitudinal cognitive trajectories in late middle-age.

Future directions for this work include: 1) examining multiple self-reported measures and the mediating effects of cardiovascular disease and other medical comorbidities; 2) determining the effect of these behaviors on risk of Alzheimer’s disease by examining imaging and cerebrospinal fluid markers of beta-amyloid, phosphorylated tau, and neurodegeneration as outcomes; and 3) determining whether health behaviors moderate risk of cognitive decline or AD based on genetic markers of AD.

In conclusion, we found that self-reported physical activity, cognitive activity, and diet—when considered individually and together, but without inclusion of objective health measures—were not significantly associated with cognitive decline in a late middle-aged (spanning ages 45 to 79), cognitively unimpaired cohort at increased risk for AD. These findings may be important for public health messaging regarding health behaviors. It is possible that these behaviors alone may not be enough to reduce risk of cognitive decline in middle age, and that managing other modifiable risk factors such as hypertension or obesity, may be required for these behaviors to have an effect on cognition. Our future work including comorbidities, overall health behavior indices in the IT model averaging approach, and additional years of follow up, and associations with Alzheimer’s disease biomarkers, will help to address these questions further.

## Supporting information

S1 Table Adapted MIND diet questionnaire* used in the WRAP study. *Adapted from Morris, M. C., Tangney, C. C., Wang, Y., Sacks, F. M., Bennett, D. A., & Aggarwal, N. T. (2015). MIND diet associated with reduced incidence of Alzheimer's disease. *Alzheimer's & Dementia*, *11*(9), 1007–1014.
(DOCX)

S2 Table Comparison of coding schemes between Morris et al. (2015) and that used in WRAP. * Morris, M. C., Tangney, C. C., Wang, Y., Sacks, F. M., Barnes, L. L., Bennett, D. A., & Aggarwal, N. T. (2015). MIND diet slows cognitive decline with aging. *Alzheimer's & dementia*, *11*(9), 1015–1022. †Responses were converted to real numbers where possible. If the conversion didn't translate to a real number, or if the resulting number was less than zero, the response was treated as a missing value. It should also be noted that if the participant responded with ">" a number, a value of ".2" was added to the converted value, i.e. a field value of "> 1" was assigned a value of "1.2". Likewise, if a "<" was found at the beginning of the field, a value of ".2" was subtracted from the converted value, i.e. a field value of "< 1" was assigned a value of ".8". Any missing field values were not included in the "MIND Diet Score" calculation. For those participants with missing values, a linear “extrapolated sum” was computed based on the number of existing scores and total number of items. For example, if there were three responses missing, and the sum of all scores present was 6.5, then the extrapolated sum would be calculated as follows: (6.5 / 12) * 15 = 8.125. **This coding scheme was implemented due to participant reports that did not fall within any of the three specified ranges of the published criteria (Morris et al., 2015).
(DOCX)

S3 Table Demographic and clinical characteristics of participants excluded due to lack of longitudinal cognitive data. (DOCX)
