# Supplementary material for: Self-reported health behaviors and longitudinal cognitive performance in late middle age: Results from the Wisconsin Registry for Alzheimer’s Prevention
Source: PLoS One. 2020 Apr 23;15(4):e0221985. doi: 10.1371/journal.pone.0221985 (PMC7179879; doi:10.1371/journal.pone.0221985)
Supplement: S1 Table — *Adapted from Morris, M. C., Tangney, C. C., Wang, Y., Sacks, F. M., Bennett, D. A., & Aggarwal, N. T. (2015). MIND diet associated with reduced incidence of Alzheimer's disease. Alzheimer's & Dementia, 11(9), 1007–1014. (DOCX) [file pone.0221985.s001.docx]

**Table A.**

| **Question** | **Frequency** |
| --- | --- |
| 1. How many tablespoons of olive oil do you consume **per day** (including that used in salad dressings and sautéing,)? | _____ **T per day** |
| 2. How many servings of green leafy vegetables do you eat **each day**, such as spinach, kale, greens, romaine? (1c for leafy, 1/2c for cooked/raw chopped) | _____ **per day** |
| 3. How many servings (1/2c) of other types of vegetables do you eat **each day** (e.g. broccoli, carrots, peas, onions, green/red peppers, celery, string beans, tomatoes, yams, squash, eggplant) ? | _____ **per day** |
| 4. How many servings (1/2c) of berries do you eat **each week** (e.g. strawberries, blueberries, raspberries)? | _____ **per week** |
| 5. How many servings of red meat (steak, ham, roast), hamburger, hot dogs or sausages do you consume **each week**? (3 oz) | _____ **per week** |
| 6. How many servings of fish (not fried and not including shellfish) do you consume **each week**? (3 oz ) | _____ **per week** |
| 7. How many servings of chicken (not fried) do you consume **each week**? (3 oz) | _____ **per week** |
| 8. How many servings of whole fat or regular cheese or cream cheese do you eat **each week**? | _____ **per week** |
| 9. How many servings of butter or cream (half & half) do you consume **each day**? (serving = 1 T) | _____ **T per day** |
| 10. How many servings of beans (1/2c) do you consume **each week**? | _____ **per week** |
| 11. How often do you eat whole grain breads, pasta, or cereals **each day**? (1 slice bread, 3/4c pasta/cereal) | _____ **per day** |
| 12. How often do you consume sweets, candy bars, pastries, cookies or cakes **per week**? | _____ **per week** |
| 13. How many servings of nuts do you eat **each week**? (handful or 1/4 - 1/3c) | _____ **per week** |
| 14. How many times **per week** do you consume food from a fast food restaurant such as McDonald’s, Burger King, Denny’s, Domino’s, Popeyes, Kentucky Fried Chicken? | _____ **per week** |
| 15. How many servings of alcohol (5 oz wine, 12 oz beer, 1 oz hard liquor) do you drink **each day**? | _____ **per day** |

**Table B.**

| Coding scheme from Morris et al. (2015)* | | | | Revised coding scheme for the WRAP study | |
| --- | --- | --- | --- | --- | --- |
|  | Point values assigned | | |  | Differences in coding† |
|  | 0 | .5 | 1 |  |  |
| Green leafy vegetables | ≤2 servings/wk | >2 to <6 servings/wk | ≥6 servings/wk | Green leafy vegetables | Servings per day X 7; serving size = 1 cup for raw, ½ cup for cooked |
| Other Vegetables | ≤5 servings/wk | 5 to <7 wk | ≥1 serving/day | Other vegetables | Servings per day X 7; serving size = ½ cup |
| Berries | <1 serving/wk | 1/wk | ≥2 servings/wk | Berries | Serving size = ½ cup; includes blueberries, raspberries and strawberries. Any response between 1-2 per week was assigned .5 value** |
| Nuts | <1/mo | 1/mo to <5/wk | ≥5 servings/wk | Nuts | Servings per week X 4.33 = servings per month. Serving size = 1 handful or 1/4 to 1/3 cup. |
| Olive Oil | Not primary oil |  | Primary oil used | Olive oil | Responses were compared with “Butter and Cream” responses; if butter and cream was greater than olive oil consumption, a value of 0 was assigned for olive oil. If olive oil consumption was greater than butter and cream, a value of 1 for olive oil was assigned. *Note: neither questionnaire asked about other oils such as canola, peanut, vegetable, etc.* |
| Butter, margarine | >2 T/d | 1–2/d | <1 T/d | Butter, cream (half and half) | Margarine is not referenced; asks instead about butter and cream or half and half; serving size = 1 tbs |
| Cheese | 7 + servings/wk | 1–6/wk | <1 serving/wk | Cheese | Described as “whole fat or regular cheese or cream cheese”. Serving size not specified. Anything between 1 and 7 servings/week was assigned a .5 value** |
| Whole grains | <1 serving/d | 1–2/d | ≥3 servings/d | Whole grains | Serving = 1 slice of bread or ¾ cup pasta/cereal. Anything between 1 and 3 servings per day was assigned a .5 value** |
| Fish (not fried) | Rarely | 1–3/mo | ≥1 meals/wk | Fish (not fried) | WRAP interpreted “rarely” to mean less than 1x/month. Servings (3 oz) per week was multiplied by 4.33 to obtain servings per month. Excludes fried fish and shellfish. Any number greater than 1 serving per month and less than one meal per week was assigned .5 value** |
| Beans | <1 meal/wk | 1–3/wk | >3 meals/wk | Beans | WRAP refers to “servings” and equates to “meals.” Serving = ½ cup |
| Poultry (not fried) | <1 meal/wk | 1/wk | ≥2 meals/wk | Chicken (not fried) | Servings in WRAP = “meals”. Referred to as “chicken” not “poultry”. Any response between 1 and 2 servings per week was assigned a .5 value** |
| Red meat and products | 7 + meals/wk | 4–6/wk | <4 meals/wk | Red meat and products | Referred to as “servings” (3 oz) instead of “meals.” Anything between 4 and 7 was assigned a .5 value.** |
| Fast fried foods | 4 + times/wk | 1–3/wk | <1 time/wk | Food from a fast food restaurant | Worded as “*How many times* ***per week*** *do you consume food from a fast food restaurant such as McDonald’s, Burger King, Denny’s, Domino’s, Popeyes, Kentucky Fried Chicken?”* Anything between 1 and 4 times per week was assigned a .5 value.** |
| Pastries and sweets | 7 + servings/wk | 5–6/wk | <5 servings/wk | Pastries and sweets | Anything between 5 and 7 times per week was assigned a .5 value** |
| Wine | >1 glass/d or never | 1/mo–6/wk | 1 glass/d | Alcohol/day | Equated ‘glasses of wine’ with ‘servings of alcohol’. Servings per day = 1, a value of 1.0 was assigned. If number of servings was greater than one or exactly 0, a value of 0 was assigned; otherwise a value of .5 was assigned.** |
