# Supplementary material for: Self-reported health behaviors and longitudinal cognitive performance in late middle age: Results from the Wisconsin Registry for Alzheimer’s Prevention
Source: PLoS One. 2020 Apr 23;15(4):e0221985. doi: 10.1371/journal.pone.0221985 (PMC7179879; doi:10.1371/journal.pone.0221985)
Supplement: S2 Table — * Morris, M. C., Tangney, C. C., Wang, Y., Sacks, F. M., Barnes, L. L., Bennett, D. A., & Aggarwal, N. T. (2015). MIND diet slows cognitive decline with aging. Alzheimer's & dementia, 11(9), 1015–1022. †Responses were converted to real numbers where possible. If the conversion didn't translate to a real number, or if the resulting number was less than zero, the response was treated as a missing value. It should also be noted that if the participant responded with ">" a number, a value of ".2" was added to the converted value, i.e. a field value of "> 1" was assigned a value of "1.2". Likewise, if a "<" was found at the beginning of the field, a value of ".2" was subtracted from the converted value, i.e. a field value of "< 1" was assigned a value of ".8". Any missing field values were not included in the "MIND Diet Score" calculation. For those participants with missing values, a linear “extrapolated sum” was computed based on the number of existing scores and total number of items. For example, if there were three responses missing, and the sum of all scores present was 6.5, then the extrapolated sum would be calculated as follows: (6.5 / 12) * 15 = 8.125. **This coding scheme was implemented due to participant reports that did not fall within any of the three specified ranges of the published criteria (Morris et al., 2015). (DOCX) [file pone.0221985.s002.docx]

**Table B.**

| Coding scheme from Morris et al. (2015)* | | | | Revised coding scheme for the WRAP study | |
| --- | --- | --- | --- | --- | --- |
|  | Point values assigned | | |  | Differences in coding† |
|  | 0 | .5 | 1 |  |  |
| Green leafy vegetables | ≤2 servings/wk | >2 to <6 servings/wk | ≥6 servings/wk | Green leafy vegetables | Servings per day X 7; serving size = 1 cup for raw, ½ cup for cooked |
| Other Vegetables | ≤5 servings/wk | 5 to <7 wk | ≥1 serving/day | Other vegetables | Servings per day X 7; serving size = ½ cup |
| Berries | <1 serving/wk | 1/wk | ≥2 servings/wk | Berries | Serving size = ½ cup; includes blueberries, raspberries and strawberries. Any response between 1-2 per week was assigned .5 value** |
| Nuts | <1/mo | 1/mo to <5/wk | ≥5 servings/wk | Nuts | Servings per week X 4.33 = servings per month. Serving size = 1 handful or 1/4 to 1/3 cup. |
| Olive Oil | Not primary oil |  | Primary oil used | Olive oil | Responses were compared with “Butter and Cream” responses; if butter and cream was greater than olive oil consumption, a value of 0 was assigned for olive oil. If olive oil consumption was greater than butter and cream, a value of 1 for olive oil was assigned. *Note: neither questionnaire asked about other oils such as canola, peanut, vegetable, etc.* |
| Butter, margarine | >2 T/d | 1–2/d | <1 T/d | Butter, cream (half and half) | Margarine is not referenced; asks instead about butter and cream or half and half; serving size = 1 tbs |
| Cheese | 7 + servings/wk | 1–6/wk | <1 serving/wk | Cheese | Described as “whole fat or regular cheese or cream cheese”. Serving size not specified. Anything between 1 and 7 servings/week was assigned a .5 value** |
| Whole grains | <1 serving/d | 1–2/d | ≥3 servings/d | Whole grains | Serving = 1 slice of bread or ¾ cup pasta/cereal. Anything between 1 and 3 servings per day was assigned a .5 value** |
| Fish (not fried) | Rarely | 1–3/mo | ≥1 meals/wk | Fish (not fried) | WRAP interpreted “rarely” to mean less than 1x/month. Servings (3 oz) per week was multiplied by 4.33 to obtain servings per month. Excludes fried fish and shellfish. Any number greater than 1 serving per month and less than one meal per week was assigned .5 value** |
| Beans | <1 meal/wk | 1–3/wk | >3 meals/wk | Beans | WRAP refers to “servings” and equates to “meals.” Serving = ½ cup |
| Poultry (not fried) | <1 meal/wk | 1/wk | ≥2 meals/wk | Chicken (not fried) | Servings in WRAP = “meals”. Referred to as “chicken” not “poultry”. Any response between 1 and 2 servings per week was assigned a .5 value** |
| Red meat and products | 7 + meals/wk | 4–6/wk | <4 meals/wk | Red meat and products | Referred to as “servings” (3 oz) instead of “meals.” Anything between 4 and 7 was assigned a .5 value.** |
| Fast fried foods | 4 + times/wk | 1–3/wk | <1 time/wk | Food from a fast food restaurant | Worded as “*How many times* ***per week*** *do you consume food from a fast food restaurant such as McDonald’s, Burger King, Denny’s, Domino’s, Popeyes, Kentucky Fried Chicken?”* Anything between 1 and 4 times per week was assigned a .5 value.** |
| Pastries and sweets | 7 + servings/wk | 5–6/wk | <5 servings/wk | Pastries and sweets | Anything between 5 and 7 times per week was assigned a .5 value** |
| Wine | >1 glass/d or never | 1/mo–6/wk | 1 glass/d | Alcohol/day | Equated ‘glasses of wine’ with ‘servings of alcohol’. Servings per day = 1, a value of 1.0 was assigned. If number of servings was greater than one or exactly 0, a value of 0 was assigned; otherwise a value of .5 was assigned.** |
