# Supplementary material for: Self-reported health behaviors and longitudinal cognitive performance in late middle age: Results from the Wisconsin Registry for Alzheimer’s Prevention
Source: PLoS One. 2020 Apr 23;15(4):e0221985. doi: 10.1371/journal.pone.0221985 (PMC7179879; doi:10.1371/journal.pone.0221985)
Supplement: S3 Table — (DOCX) [file pone.0221985.s003.docx]

**S3. Demographic and clinical characteristics of excluded participants due to lack of longitudinal cognitive data**

|  | **Not in study**  (mean (sd) or %) | **In study**  (mean (sd) or %) | **p** |
| --- | --- | --- | --- |
|  |  |  |  |
| n | 52 | 828 |  |
| Baseline Age | 55.48 (6.78) | 57.75 (6.37) | 0.027 |
| Years of Follow-up | 6.71 (3.87) | 6.29 (1.93) | 0.076 |
| Sex (female) (%) | 32 (61.5) | 560 ( 67.6) | 0.364 |
| APOE-E4 positive (%) | 18 (41.9) | 312 ( 37.7) | 0.63 |
| WRAT- Reading | 105.08 (10.28) | 105.72 (9.29) | 0.804 |
| CES-D | 7.37 (8.06) | 6.61 (6.72) | 0.961 |
| race = white (%) | 36 (83.7) | 812 ( 98.2) | <0.001 |
| Years of Education | 16.32 (2.64) | 16.22 (2.75) | 0.819 |
| Systolic Blood Pressure | 136.71 (122.78) | 124.19 (15.90) | 0.1 |
| Diastolic Blood Pressure | 92.38 (128.44) | 74.11 (9.59) | 0.4 |
| Total Cholesterol | 191.12 (35.04) | 200.30 (37.73) | 0.033 |
| Body Mass Index | 29.45 (7.17) | 28.70 (6.07) | 0.342 |
| Wasit-hip-ratio | 0.87 (0.08) | 0.86 (0.10) | 0.645 |
| MMSE | 29.78 (0.44) | 29.43 (0.91) | 0.276 |
| AVLT Total | 48.73 (10.75) | 51.12 (8.39) | 0.147 |
| Logical Memory Delayed | 24.22 (8.56) | 26.22 (6.86) | 0.602 |
| Digit Symbol Coding | 56.00 (10.77) | 57.48 (10.17) | 0.915 |
